# Chemotherapy for Older Adults with Locally Advanced or Metastatic Pancreatic Ductal Adenocarcinoma: A Systematic Review and Meta-Analysis

**DOI:** 10.3390/jcm15062254

**Published:** 2026-03-16

**Authors:** Dong Woo Shin, Ji Su Ahn, Hyunjoo Song, Sung-Hoon Moon, Jong-chan Lee

**Affiliations:** 1 Department of Internal Medicine, Hallym University College of Medicine, Hallym University Sacred Heart Hospital, Anyang 14068, Republic of Korea; delight0618@naver.com (D.W.S.); endomoon@hallym.or.kr (S.-H.M.); 2 Department of Internal Medicine, Seoul National University Bundang Hospital, Seongnam 13620, Republic of Korea; jisuahn0103@gmail.com; 3 School of Computer Science and Engineering, Soongsil University, Seoul 06978, Republic of Korea; hsong@ssu.ac.kr

**Keywords:** pancreatic cancer, elderly, chemotherapy, overall survival, progression-free survival, FOLFIRINOX, nab-paclitaxel, meta-analysis

## Abstract

**Background:** Treatment decisions for older adults with locally advanced or metastatic pancreatic ductal adenocarcinoma (PDAC) often rely on heterogeneous observational evidence and clinical judgment regarding survival benefits, regimen intensity, and tolerability. **Methods:** We systematically searched Embase, PubMed, and Scopus from inception to 30 March 2025, for studies reporting overall survival (OS) and/or progression-free survival (PFS) in older adults with advanced PDAC receiving systemic chemotherapy, as well as age-stratified outcomes among chemotherapy-treated patients. Hazard ratios (HRs) with 95% confidence intervals (CIs) were primarily extracted from multivariable-adjusted analyses. In cases without reported HRs, estimates were derived from summary statistics or Kaplan–Meier curves. The review protocol was registered in PROSPERO (CRD420261292913). **Results:** A total of 40 predominantly retrospective studies were included. Chemotherapy was associated with improved OS compared to best supportive care in older adults (9 studies; HR 0.46, 95% CI 0.39–0.54; *I*^2^ = 18%). Among chemotherapy-treated patients, OS (34 studies; HR 1.00, 95% CI 0.99–1.02; *I*^2^ = 23%) and PFS (11 studies; HR 0.96, 95% CI 0.86–1.07; *I*^2^ = 10%) did not differ by age. Combination chemotherapy demonstrated superior OS (13 studies; HR 0.66, 95% CI 0.54–0.80; *I*^2^ = 86%) with substantial heterogeneity and PFS (7 studies; HR 0.63, 95% CI 0.53–0.74; *I*^2^ = 30%) compared to monotherapy. FOLFIRINOX and gemcitabine plus nab-paclitaxel demonstrated comparable OS (8 studies; HR 0.98, 95% CI 0.90–1.05; *I*^2^ = 60%) and PFS (2 studies; HR 0.97, 95% CI 0.92–1.02; *I*^2^ = 0%). **Conclusions:** Among carefully selected older adults with advanced PDAC, chemotherapy was associated with improved survival compared to supportive care. Chronological age did not predict outcomes, highlighting the need for geriatric-informed prospective trials.

## 1. Introduction

Pancreatic ductal adenocarcinoma (PDAC) remains one of the most lethal malignancies, with most patients diagnosed at an unresectable stage (locally advanced or metastatic) and primarily treated with systemic therapy [1,2,3]. As the global population ages, PDAC is increasingly diagnosed in older adults, creating a critical unmet need for age-inclusive evidence to guide treatment selection and intensity [4,5]. For older adults with advanced PDAC, critical uncertainties persist regarding: (1) the survival benefit of chemotherapy versus best supportive care (BSC) alone; (2) combination chemotherapy versus monotherapy; and (3) the comparative effectiveness of FOLFIRINOX versus gemcitabine plus nab-paclitaxel [6,7].

Chronological age is an imperfect predictor of treatment tolerance, as physiological reserve varies widely among older adults [8]. Frailty, multimorbidity, polypharmacy, and diminished physiological reserve constrain dose intensity, heighten toxicity risk, and amplify competing mortality [9]. However, older adults are underrepresented in pivotal trials, and those enrolled are highly selected fit individuals, limiting generalizability to real-world practice [10]. Consequently, clinicians must extrapolate from younger trial populations despite concerns about toxicity, dose reductions, and treatment discontinuation in older adults. Most evidence informing chemotherapy use in older adults with advanced PDAC comes from observational studies, predominantly retrospective [11,12]. Non-randomized treatment allocation in these studies introduces substantial confounding by indication and selection bias. Evidence is further fragmented by inconsistent age cutoffs (≥65, ≥70, or ≥75 years), heterogeneous disease settings (locally advanced vs. metastatic), and diverse treatment regimens (monotherapy, combination chemotherapy, and intensive multi-agent protocols). These methodological inconsistencies complicate evidence synthesis and limit translation into clinical practice guidelines. Moreover, key clinical comparisons—chemotherapy versus BSC, age-stratified outcomes, combination versus monotherapy, and regimen effectiveness—have not been synthesized using a consistent analytical approach with standardized survival endpoints. While prior meta-analyses have evaluated chemotherapy outcomes in older adults with PDAC, important gaps remain. Many earlier reviews predated widespread use of contemporary combination regimens and did not systematically compare combination versus monotherapy strategies or examine the prognostic relevance of chronological age among treated patients. The present study addresses these limitations by providing updated pooled hazard ratios and incorporating modern systemic regimens with stage-inclusive and age-stratified analyses.

To address these gaps, we conducted a systematic review and meta-analysis following Preferred Reporting Items for Systematic Reviews and Meta-Analyses (PRISMA) 2020 guidelines and the Meta-analysis Of Observational Studies in Epidemiology (MOOSE) reporting standards for observational studies. We prespecified four clinically relevant comparisons: (1) chemotherapy versus BSC in older adults; (2) older versus younger patients receiving chemotherapy; (3) combination versus monotherapy in older adults; and (4) FOLFIRINOX versus gemcitabine plus nab-paclitaxel in older adults. We aimed to establish a clinically actionable, evidence-based framework for treatment decisions—including whether to initiate chemotherapy, optimal intensity, and regimen selection—in older adults with advanced PDAC.

## 2. Materials and Methods

### 2.1. Protocol and Reporting Standards

**Figure 1:**
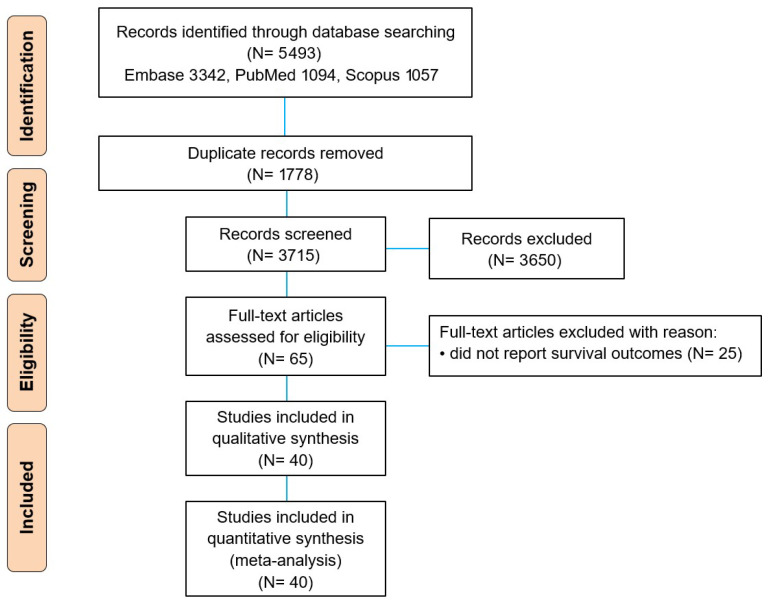
PRISMA flow diagram of study selection.

This systematic review and meta-analysis was conducted in accordance with PRISMA 2020 guidelines (Figure 1). Given that the evidence base consisted predominantly of observational studies, our reporting was also guided by MOOSE recommendations, where applicable. The review questions and analytical plan, including prespecified comparisons and outcomes, were established prior to the selection of studies, and the review protocol was registered in PROSPERO (CRD420261292913). Since this study synthesized published aggregate data without access to individual patient identifiers, institutional review board approval and informed consent were not required.

### 2.2. Data Sources and Search Strategy

We conducted a systematic search of Embase, PubMed, and Scopus from database inception through 30 March 2025. The search strategy combined controlled vocabulary and free-text terms covering: (1) pancreatic cancer/pancreatic ductal adenocarcinoma; (2) older age or age-stratified comparisons; and (3) systemic chemotherapy, including FOLFIRINOX and nab-paclitaxel-based regimens. The core search string, adapted to each database syntax, was (Pancreatic cancer OR pancreatic ductal adenocarcinoma) AND (elderly OR old OR older OR geriatric OR aged OR younger) AND (FOLFIRINOX OR nab-paclitaxel OR abraxane).

### 2.3. Eligibility Criteria

Studies were eligible for inclusion if they reported overall survival (OS) and/or progression-free survival (PFS) in elderly patients with locally advanced or metastatic PDAC, including studies that provided age-stratified outcome comparisons among patients treated with systemic chemotherapy. Both prospective and retrospective clinical studies were eligible. Using a PICOS framework: Population—locally advanced or metastatic pancreatic cancer; Intervention/exposure—chemotherapy or specific regimens; Comparators—BSC, younger age group, monotherapy, or alternative regimens; Outcomes—OS and/or PFS; Study types—prospective or retrospective clinical studies. Given the absence of a universally accepted definition of “elderly” in PDAC literature, studies employing age cutoffs of ≥65, ≥70, or ≥75 years were included.

Inclusion criteria were: (1) adult patients with unresectable locally advanced or metastatic PDAC treated with systemic chemotherapy; (2) a study-defined threshold for elderly age or extractable age-stratified outcome data; and (3) sufficient information to extract hazard ratios (HRs) with 95% confidence intervals (CIs), or availability of Kaplan–Meier curves or summary statistics permitting HR estimation using established reconstruction methods.

Exclusion criteria were: (1) studies enrolling predominantly resectable disease, non-pancreatic periampullary malignancies, or histologies other than PDAC; (2) insufficient data to derive or extract HRs for time-to-event outcomes; (3) non-original research reports (conference abstracts without full-text publications, narrative reviews, editorials, letters, or case reports); (4) unavailability of full-text articles despite retrieval attempts; and (5) articles published in languages other than English.

### 2.4. Study Selection Process

After duplicate removal, two reviewers (DWS, JSA) independently screened titles/abstracts and assessed full texts. Discrepancies were resolved by consensus, with third-reviewer adjudication if required. Reasons for full-text exclusion were documented (Figure 1). When multiple publications reported overlapping cohorts, we included only one report per cohort per comparison (prioritizing the most complete follow-up and/or the most fully adjusted HR). Subgroup/secondary analyses from the same parent trial were not double counted.

### 2.5. Data Extraction and Operational Definitions

Two reviewers independently extracted study-level data using a standardized data extraction form. Extracted variables included: first author and publication year, country of origin, study design, sample size, age threshold defining elderly status, age-stratified patient denominators, sex distribution, Eastern Cooperative Oncology Group performance status, disease stage (locally advanced vs. metastatic), treatment regimens administered, and time-to-event outcomes (OS and PFS with corresponding HRs and 95% CIs). When both adjusted and unadjusted effect estimates were available, adjusted HRs were preferentially extracted.

### 2.6. Outcomes and Prespecified Comparative Questions

The primary outcome was OS; PFS was the secondary outcome. The effect measure was the HR with 95% CI comparing time-to-event outcomes between groups. Quantitative syntheses were performed for the following prespecified comparisons: (1) chemotherapy versus BSC in older adults (OS); (2) older versus younger adults receiving chemotherapy (OS and PFS); (3) combination chemotherapy versus monotherapy in older adults (OS and PFS); and (4) FOLFIRINOX versus gemcitabine plus nab-paclitaxel in older adults (OS and PFS). To prevent double counting, each study contributed data to only one comparison per outcome, even when multi-arm designs provided data relevant to multiple comparisons.

### 2.7. Derivation of Time-to-Event Estimates

HRs and 95% CIs were extracted directly when explicitly reported in the original publications. When HRs were not provided, log(HR) and its standard error were derived from available summary statistics (e.g., log-rank *p* values, numbers of events, or other time-to-event summaries) using established methods (e.g., Tierney et al.) [13]. For reconstructed estimates, conservative assumptions were applied when reporting was incomplete (e.g., limited numbers-at-risk information), and such estimates were flagged during extraction for prespecified sensitivity analyses.

### 2.8. Statistical Methods

For each comparison, log-transformed HRs were pooled using an inverse-variance weighted random-effects model (DerSimonian–Laird method) to account for anticipated between-study heterogeneity. Statistical heterogeneity was quantified using the *I*^2^ statistic and interpreted in conjunction with clinical and methodological diversity across studies. When substantial heterogeneity was observed and reporting permitted, we explored potential sources using prespecified stratifications (e.g., study-defined elderly cutoff, disease setting, and effect estimate type such as multivariable-adjusted versus unadjusted/reconstructed). Prespecified sensitivity analyses to examine robustness included influence analyses (leave-one-out meta-analyses), restriction to multivariable-adjusted HRs, and exclusion of reconstructed estimates; these were conducted only when sufficient data and reporting quality permitted reliable implementation. Publication bias and small-study effects were evaluated through visual inspection of funnel plots for each major comparison when sufficient studies were available. All statistical analyses were performed using SPSS version 25.0 (IBM Corp., Armonk, NY, USA) and R version 3.2.3 (The R Foundation for Statistical Computing, Vienna, Austria). Two-sided *p* values < 0.05 were considered statistically significant.

## 3. Results

### 3.1. Study Selection

The systematic search identified 5493 records across three databases: Embase (*n* = 3342), PubMed (*n* = 1094), and Scopus (*n* = 1057). Following removal of 1778 duplicate records, 3715 records underwent title and abstract screening. Sixty-five full-text articles were subsequently retrieved and assessed for eligibility against predefined inclusion and exclusion criteria. Among the full-text articles assessed for eligibility, 25 were excluded due to the absence of extractable survival data. Ultimately, 40 studies met all eligibility criteria and were included in both qualitative synthesis and quantitative meta-analysis (Figure 1).

### 3.2. Characteristics of Included Studies

The majority of included studies employed retrospective designs (36 of 40 studies, 90%), with four prospective cohort studies. Geographically, studies originated from 12 countries, with the most frequent contributions from the United States and Japan (8 studies each), followed by Korea (5 studies). Age thresholds for older adults varied across studies, with ≥65 years being most common (18 studies, 45.0%), followed equally by ≥70 years and ≥75 years (11 studies each, 27.5%). Reported disease characteristics and treatment regimens were heterogeneous, encompassing BSC, gemcitabine-based monotherapy, combination chemotherapy regimens (including FOLFIRINOX [FLX] and gemcitabine plus nab-paclitaxel [GNP]), and other systemic therapies. Detailed study characteristics are summarized in Table 1.

### 3.3. Chemotherapy Versus Best Supportive Care in Older Patients (Overall Survival)

**Figure 2:**
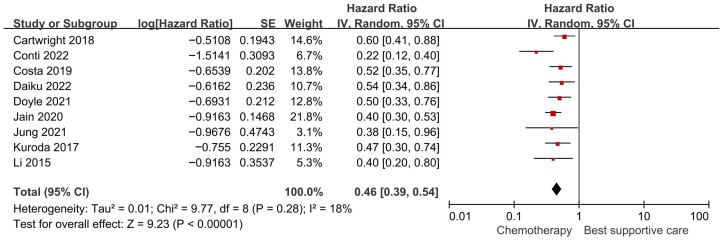
Forest plot of overall survival comparing systemic chemotherapy versus best supportive care in older adults with advanced pancreatic ductal adenocarcinoma.

Nine studies provided comparative data on chemotherapy versus BSC in older adults with advanced PDAC. Meta-analysis demonstrated that chemotherapy was associated with a lower hazard of death compared with BSC (pooled HR 0.46, 95% CI 0.39–0.54; *p* < 0.001). Statistical heterogeneity was low (*I*^2^ = 18%), suggesting consistency of the survival benefit across studies (Figure 2; Table 2). Given the predominantly retrospective and non-randomized nature of the included studies, this observed association should not be interpreted as definitive evidence of a causal treatment effect.

### 3.4. Age-Stratified Outcomes in Patients Receiving Chemotherapy

#### 3.4.1. Overall Survival

**Figure 3:**
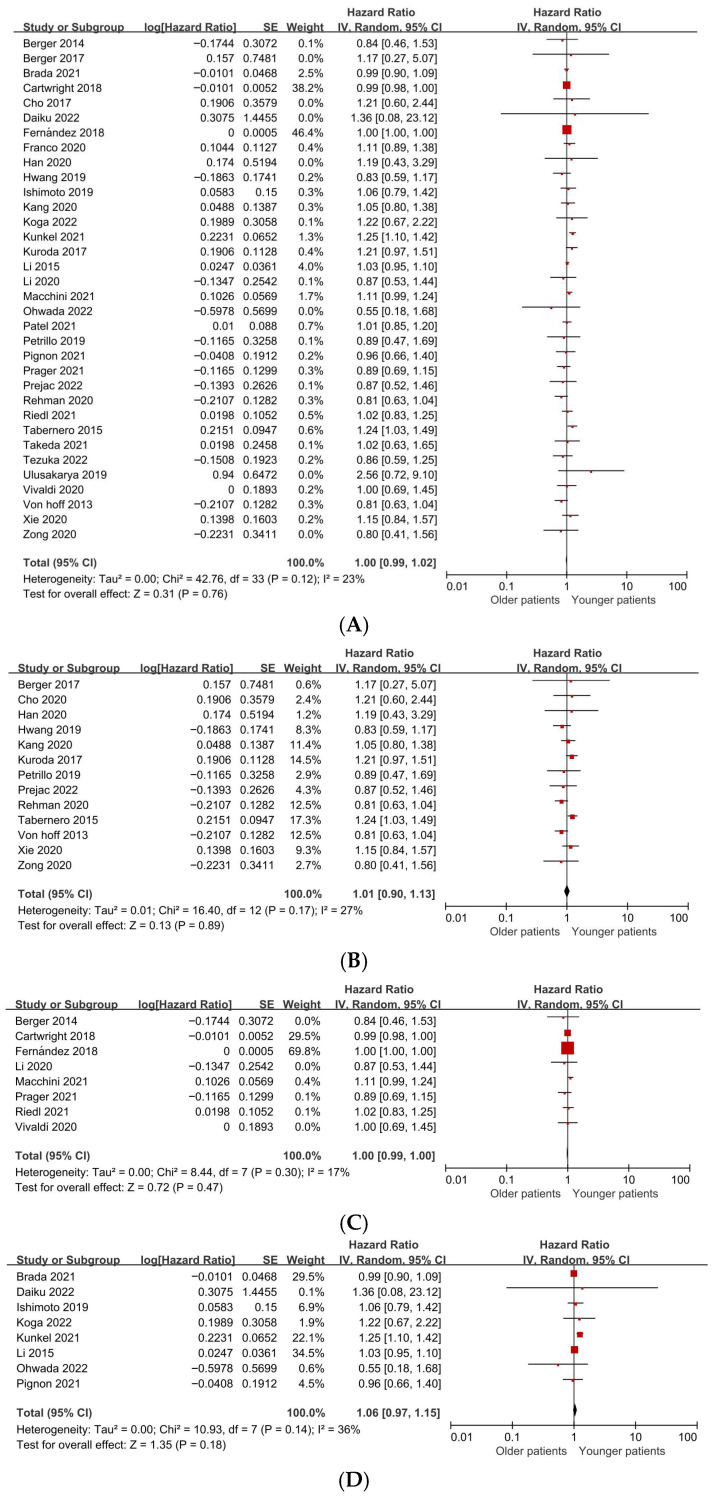

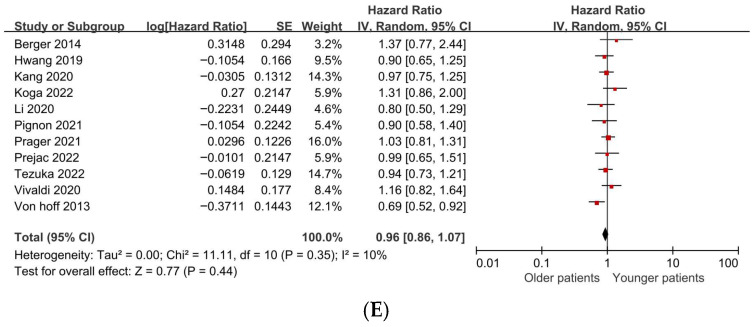
(**A**). Forest plot of age-stratified outcomes among chemotherapy-treated patients with pancreatic ductal adenocarcinoma (overall survival). (**B**). Forest plot of age-stratified overall survival among chemotherapy-treated patients with pancreatic ductal adenocarcinoma (≥65-year cutoff). (**C**). Forest plot of age-stratified overall survival among chemotherapy-treated patients with pancreatic ductal adenocarcinoma (≥70-year cutoff). (**D**). Forest plot of age-stratified overall survival among chemotherapy-treated patients with pancreatic ductal adenocarcinoma (≥75-year cutoff). (**E**). Forest plots of age-stratified outcomes among chemotherapy-treated patients with pancreatic ductal adenocarcinoma (progression-free survival).

Among patients selected to receive systemic chemotherapy, OS did not differ significantly between older and younger age groups. Meta-analysis of 34 studies yielded a pooled HR of 1.00 (95% CI 0.99–1.02; *p* = 0.76), with low statistical heterogeneity (*I*^2^ = 23%), indicating comparable survival outcomes across age strata when chemotherapy is administered (Figure 3A; Table 2).

#### 3.4.2. Subgroup Analysis of Overall Survival by Age Cutoff

To evaluate the impact of heterogeneous age definitions across studies, we performed subgroup analyses stratified by study-defined elderly thresholds (≥65, ≥70, and ≥75 years). Among studies defining older age as ≥65 years, the pooled hazard ratio for OS was 1.01 (95% CI 0.90–1.13; *p* = 0.89), with low-to-moderate heterogeneity (*I*^2^ = 27%) (Figure 3B). For studies using a ≥70-year cutoff, the pooled HR was 1.00 (95% CI 0.99–1.00; *p* = 0.47), with modest heterogeneity (*I*^2^ = 17%) (Figure 3C). Similarly, studies defining older age as ≥75 years demonstrated a pooled HR of 1.06 (95% CI 0.97–1.15; *p* = 0.18), with low heterogeneity (*I*^2^ = 36%) (Figure 3D).

#### 3.4.3. Progression-Free Survival

Similarly, PFS did not differ by age among chemotherapy-treated patients. Analysis of 11 studies reporting age-stratified PFS data showed a pooled HR of 0.96 (95% CI 0.86–1.07; *p* = 0.44), with low heterogeneity (*I*^2^ = 10%) (Figure 3E; Table 2).

### 3.5. Combination Chemotherapy Versus Monotherapy in Older Patients

#### 3.5.1. Overall Survival

**Figure 4:**
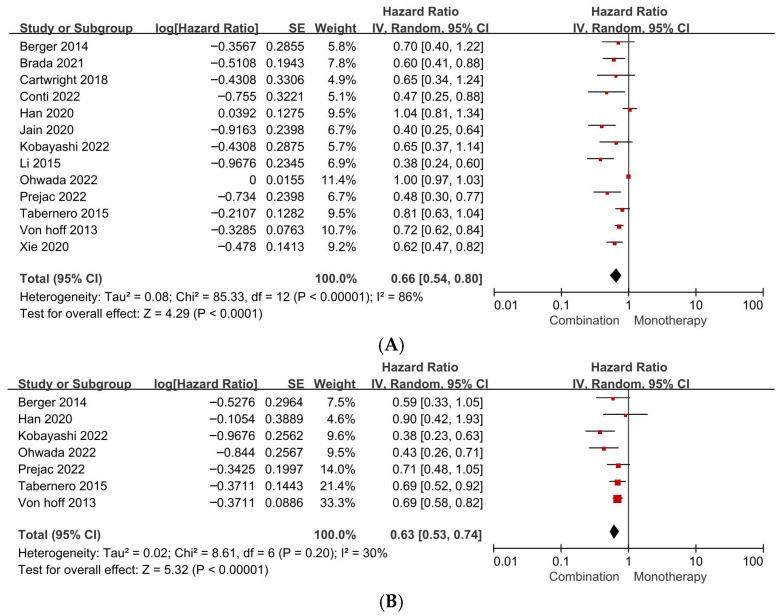
(**A**). Forest plot of survival outcomes in older adults with pancreatic ductal adenocarcinoma comparing combination chemotherapy versus monotherapy (overall survival). (**B**). Forest plot of survival outcomes in older adults with pancreatic ductal adenocarcinoma comparing combination chemotherapy versus monotherapy (progression-free survival).

In older adults, combination chemotherapy regimens were associated with significantly improved OS compared with monotherapy. Meta-analysis of 13 studies yielded a pooled HR of 0.66 (95% CI 0.54–0.80; *p* < 0.001), corresponding to a 34% reduction in the hazard of death with combination therapy. However, substantial statistical heterogeneity was observed (*I*^2^ = 86%) (Figure 4A; Table 2).

#### 3.5.2. Progression-Free Survival

Combination chemotherapy also conferred a significant PFS advantage over monotherapy in older adults. Seven studies contributed to this analysis, demonstrating a pooled HR of 0.63 (95% CI 0.53–0.74; *p* < 0.001), with lower heterogeneity (*I*^2^ = 30%) than observed for OS (Figure 4B; Table 2).

### 3.6. FOLFIRINOX Versus Gemcitabine Plus Nab-Paclitaxel in Older Patients

#### 3.6.1. Overall Survival

**Figure 5:**
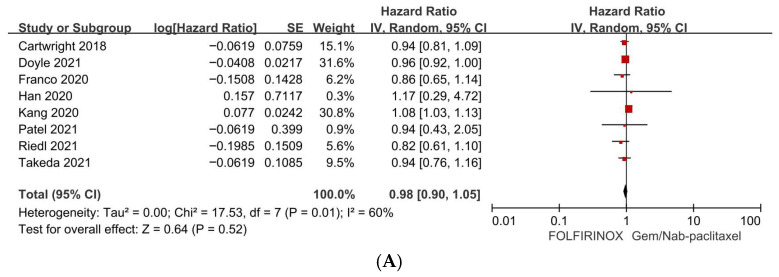

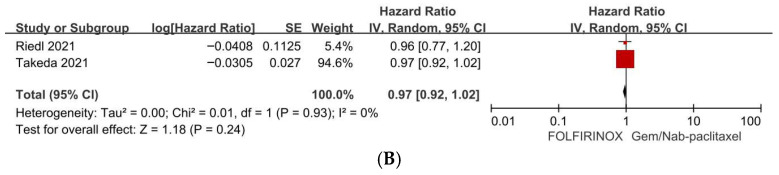
(**A**). Forest plot of survival outcomes in older adults with advanced pancreatic ductal adenocarcinoma comparing FOLFIRINOX versus gemcitabine plus nab-paclitaxel (overall survival). (**B**). Forest plot of survival outcomes in older adults with advanced pancreatic ductal adenocarcinoma comparing FOLFIRINOX versus gemcitabine plus nab-paclitaxel (progression-free survival).

Among older adults treated with intensive combination chemotherapy, OS outcomes did not differ significantly between FLX and GNP regimens. Eight studies provided comparative data, yielding a pooled HR of 0.98 (95% CI 0.90–1.05; *p* = 0.52). Moderate statistical heterogeneity was present (*I*^2^ = 60%), potentially reflecting differences in patient selection, dose modifications, and supportive care practices across studies (Figure 5A; Table 2).

#### 3.6.2. Progression-Free Survival

Evidence comparing PFS between FLX and GNP in older adults was limited to two studies. This analysis showed no significant difference in progression risk (pooled HR 0.97, 95% CI 0.92–1.02; *p* = 0.24), with no heterogeneity (*I*^2^ = 0%). However, the paucity of studies limits confidence in this estimate (Figure 5B; Table 2).

### 3.7. Publication Bias Assessment

Visual inspection of funnel plots did not demonstrate clear asymmetry for the primary comparisons (Supplementary Figure S1A–G).

## 4. Discussion

In this systematic review and meta-analysis focusing on older adults with PDAC, we synthesized contemporary evidence addressing critical and clinically relevant questions that frequently arise in clinical practice. Specifically, we examined whether systemic chemotherapy confers a significant benefit over BSC, whether chronological age serves as an independent predictor of inferior clinical outcomes, and how commonly utilized chemotherapy regimens and their intensities compare within an older patient population. Among older adults, systemic chemotherapy was associated with a 54% relative reduction in the hazard of death compared to BSC (HR 0.46, 95% CI 0.39–0.54; *I*^2^ = 18%). Among chemotherapy-treated patients, chronological age was not associated with inferior outcomes (HR 1.00, 95% CI 0.99–1.02; *I*^2^ = 23%) or PFS (HR 0.96, 95% CI 0.86–1.07; *I*^2^ = 10%). Combination chemotherapy was associated with improved survival versus monotherapy (OS: HR 0.66, 95% CI 0.54–0.80, *I*^2^ = 86%; PFS: HR 0.63, 95% CI 0.53–0.74, *I*^2^ = 30%), while FOLFIRINOX and gemcitabine plus nab-paclitaxel demonstrated comparable efficacy (OS: HR 0.98, 95% CI 0.90–1.05, *I*^2^ = 60%; PFS: HR 0.97, 95% CI 0.92–1.02, *I*^2^ = 0%).

Despite the establishment of modern treatment standards for advanced PDAC through landmark randomized trials, older and frailer patients remain consistently underrepresented in clinical research. This underrepresentation limits the external validity of trial-derived estimates when applied to routine clinical practice [54,55,56]. This evidence gap contributes to a pervasive sense of therapeutic nihilism and the age-based undertreatment of patients, particularly as the aging population continues to increase the absolute burden of PDAC among older adults [57,58,59]. Our findings advocate for a treatment paradigm that prioritizes physiological reserve and individual patient goals over chronological age alone in guiding systemic therapy decisions. This shift is essential to improve the quality of care for older adults with PDAC and to ensure that they receive the most appropriate and effective treatment tailored to their unique circumstances [4,5,60].

First, chemotherapy provides a significant survival gain over BSC in older patients with advanced PDAC. These findings are consistent with the broader literature on PDAC, which illustrates meaningful survival gains from systemic therapy in fit patients, emphasizing that the potential for life prolongation remains clinically relevant, even among older adults, when treatment is feasible [61,62]. Importantly, the BSC comparator in routine clinical practice often reflects a complex interplay of factors, including frailty, comorbidities, patient preferences, late presentations, and logistical barriers, rather than solely indicating a “no-treatment” choice. Therefore, the observed magnitude of benefit should be interpreted as an association between treatment receipt and outcomes in real-world settings, rather than implying a definitive causal effect. Because treatment allocation in these studies was non-randomized, the observed magnitude of benefit likely reflects, at least in part, confounding by indication and selection of biologically fitter older adults for systemic therapy. Additionally, immortal-time bias may have contributed to the apparent survival advantage, as patients must survive long enough to initiate chemotherapy, further limiting causal inference. Nonetheless, the consistent direction and strength of this effect provide compelling evidence that systemic therapy should be actively considered in older adults when feasible, rather than being deferred by default.

Second, chronological age alone was not associated with poorer outcomes in treated patients. Comparative studies of older and younger groups revealed similar OS, indicating no significant difference (HR 1.00) and PFS also exhibited no clinically meaningful disparity (HR 0.96). These findings yield two complementary implications: (i) they reinforce the notion that age should not be used as a sole indicator of prognosis or treatment intolerance, a perspective increasingly cautioned against in contemporary oncology [63,64]; (ii) these results may reflect selection biases inherent in observational studies: older adults receiving chemotherapy often represent a biologically fitter subset, characterized by preserved performance status, supportive social circumstances, and lower competing mortality risks [65]. This “healthy treated elderly” phenomenon is particularly relevant in PDAC, where rapid clinical decline may preclude treatment and exacerbate survivorship bias [11,12]. However, similar survival between older and younger treated patients does not imply that all older adults tolerate treatment equally well. Importantly, this finding likely reflects selection of biologically fitter older adults for systemic therapy in observational cohorts and should not be interpreted as evidence that chronological age lacks independent prognostic relevance in unselected patient populations. Thus, our findings should not be interpreted as an assurance that all older adults will fare as well as their younger counterparts; rather, they provide evidence that fit older adults can achieve outcomes comparable to younger patients when systemic therapy is appropriately administered [19,66]. These findings underscore the importance of structured assessments of vulnerability and physiological reserve in guiding therapeutic decisions for older adults with PDAC. The American Society of Clinical Oncology geriatric assessment guidelines advocate for the use of validated tools to identify functional impairments, comorbidity burdens, cognitive vulnerabilities, nutritional risks, and social support deficits, all of which can significantly influence treatment toxicity and adherence [64]. Concurrently, geriatric assessment-driven intervention strategies, such as geriatric assessment–guided intervention (GAP70+), have shown clinically meaningful reductions in severe toxicity while preserving treatment outcomes in older adults receiving systemic therapy [67]. For pancreatobiliary specialists and multidisciplinary teams, the practical integration of geriatric principles—including baseline functional screening, proactive nutritional and symptom management, medication reconciliation, and early engagement of palliative care—may prove to be as critical as the selection of the therapeutic regimen itself [67,68,69].

Third, treatment intensity is critical: combination chemotherapy is associated with improved outcomes compared to monotherapy in older adults. Specifically, combination therapy demonstrates superior OS (HR 0.66, 95% CI 0.54–0.80) and PFS (HR 0.63, 95% CI 0.53–0.74) relative to monotherapy. The substantial heterogeneity (*I*^2^ = 86%) likely reflects multiple sources of clinical and methodological diversity, including variation in regimen composition, dose attenuation or modified protocols, line of therapy (first-line vs. later-line), and differential selection of fitter older adults for combination regimens. Such variability limits the reliability of the pooled estimate and underscores that the observed survival advantage should be interpreted cautiously rather than as definitive evidence supporting combination therapy for all older patients. This finding is biologically plausible due to the multi-agent synergy that underlies efficacy in PDAC and aligns with the broader first-line treatment landscape, where multi-agent regimens have shown enhanced disease control in selected populations. However, translating this into clinical practice for older adults necessitates a nuanced approach. Combination therapy is not merely a binary choice but rather a spectrum that includes considerations of dose attenuation, schedule modification, and step-up strategies (e.g., initiating with a doublet and escalating based on tolerance) [25,70]. Such personalized approaches are particularly relevant in PDAC, where symptom burden—including pain, cachexia, and cholestasis—alongside the risk of infection and rapid functional decline, influences chemotherapy tolerance [71,72]. Consequently, our pooled estimates should be interpreted as supporting combination therapy for appropriately selected older patients, while also emphasizing the importance of individualized dosing and early discontinuation thresholds that align with patient goals. Importantly, the substantial heterogeneity observed in this analysis limits the strength of conclusions that can be drawn regarding treatment intensity, and the pooled effect should not be interpreted as definitive evidence favoring combination therapy for all older patients.

Fourth, among the commonly utilized first-line multi-agent regimens, FOLFIRINOX and gemcitabine plus nab-paclitaxel appear broadly comparable in older adults regarding survival endpoints. Pooled analyses indicate that OS does not significantly differ between FOLFIRINOX and gemcitabine plus nab-paclitaxel (HR 0.98, 95% CI 0.90–1.05), and PFS shows a similar trend in the limited available data (HR 0.97, 95% CI 0.92–1.02). These findings align with contemporary comparative evidence suggesting that survival differences between these regimens may be modest and context-dependent, with the choice of regimen often influenced by toxicity profiles, logistical considerations, and patient-specific vulnerabilities [45,73]. In older adults, regimen selection should account for irinotecan-associated diarrhea and neutropenia, oxaliplatin-associated neuropathy, and taxane-associated neuropathy and myelosuppression, as these toxicities can have meaningful functional consequences in this population [7]. Consequently, an “equipoise-informed” approach—selecting among reasonable options based on comorbidities, neuropathy risk, history of biliary stenting or infections, baseline nutritional status, and patient priorities—may prove most appropriate [74,75].

Several limitations warrant careful consideration. Most of the included studies were retrospective, making them susceptible to confounding by indication, immortal-time bias, and incomplete adjustment for essential geriatric determinants, such as frailty, cognitive function, and social support. Additionally, definitions of “elderly” varied across studies, and treatment regimens exhibited considerable heterogeneity concerning dose intensity, supportive measures, and line of therapy. Inconsistent reporting of toxicity, treatment discontinuation, and patient-reported outcomes further limits our ability to accurately quantify the trade-offs that are particularly salient for older patients. We acknowledge that unresectable locally advanced and metastatic PDAC represent biologically distinct entities with different prognostic profiles. However, because most included studies reported these populations collectively as advanced disease, stage-restricted meta-analytic comparisons were not consistently feasible. Furthermore, heterogeneity in the combination therapy analysis may have been amplified by differences in dose intensity, supportive care practices, and institutional treatment preferences, factors that were incompletely reported across studies. Moreover, comparisons between regimens may be particularly prone to selection bias, as clinicians may preferentially prescribe one regimen over another based on unmeasured vulnerabilities, including neuropathy risk, renal reserve, and caregiver availability. Finally, advances in supportive care and evolving standards of systemic therapy over the study period may impact the generalizability of our findings to current clinical practice, especially given the increasing adoption of modified regimens and novel combinations.

Despite these limitations, our synthesis provides pragmatic and clinically actionable insights. The comprehensive evidence supports three overarching principles: (1) chemotherapy should be actively considered for older adults with PDAC rather than withheld solely based on age; (2) fit older patients can achieve outcomes comparable to those of younger patients when treated, emphasizing the importance of avoiding age-based therapeutic nihilism; and (3) when feasible, combination therapy offers survival advantages over monotherapy, with regimen selection among multi-agent options needing to be individualized based on patient vulnerability, toxicity profiles, and specific goals of care. Future research should prioritize prospective, geriatric-informed trials and real-world comparative effectiveness studies that incorporate frailty metrics, functional endpoints, and patient-reported outcomes to better delineate the optimal balance between longevity and quality of life for older adults with PDAC. Ultimately, improving outcomes in this population will require not only careful regimen selection but also the systematic integration of geriatric assessments and meticulous pancreatobiliary supportive care throughout the treatment continuum.

## 5. Conclusions

Systemic chemotherapy was associated with improved survival in selected older patients with advanced PDAC. Combination therapy was associated with better survival than monotherapy, although this finding should be interpreted cautiously given substantial heterogeneity and the observational nature of the data.

## Figures and Tables

**Table 1:** Baseline characteristics of the included studies.

Study (Year)	Country	Design	N	Age (yrs)	Age Cut-Off for Older Group (yrs)	Older/Younger (*n*)	M/F	ECOG 0–1/2–3	FLX/GNP/Gem/Other/BSC
Berger (2014) [14]	Germany	R	53	73	70	35/16	26/27	32/21	4/0/22/27/0
Berger (2017) [15]	Germany	R	88	56	65	15/73	57/31	85/3	88/0/0/0/0
Brada (2021) [16]	The Netherlands	R	422	68	65	260/162	218/204	313/66	252/33/41/9/87
Cartwright (2018) [17]	USA	R	486	66	70	166/320	267/219	389/73	159/255/72/0/0
Cho (2017) [18]	Republic of Korea	R	81	65	65	NR/NR	37/44	81/0	0/81/0/0/0
Conti (2022) [19]	France	R	159	80	70	159/0	76/83	NR/NR	12/23/49/15/60
Costa (2019) [20]	Brazil	R	196	73	65	196/0	98/98	132/64	35/6/77/56/22
Daiku (2022) [21]	Japan	R	963	70	75	276/687	561/402	NR/NR	0/20/100/NR/124
Doyle (2021) [22]	USA	R	665	75	75	353/312	358/307	NR/NR	67/200/0/0/363
Fernández (2018) [23]	Spain	R	210	65	70	53/157	127/83	149/33	0/210/0/0/0
Franco (2021) [24]	Spain	R	119	63	70	32/87	68/51	103/16	59/60/0/0/0
Han (2020) [25]	Republic of Korea	R	104	69	65	104/0	71/33	NR/NR	25/34/45/0/0
Hwang (2019) [26]	Republic of Korea	R	203	62	65	77/126	116/87	195/8	0/203/0/0/0
Ishimoto (2019) [27]	Japan	R	27	70	75	9/18	15/12	27/0	0/27/0/0/0
Jain (2020) [28]	USA	R	473	73	65	473/0	243/230	340/75	19/31/NR/38/121
Jung (2021) [29]	Republic of Korea	R	167	74	70	167/0	78/89	81/86	3/12/18/3/131
Kang (2018) [30]	Republic of Korea	R	308	62	65	110/198	191/117	302/6	159/149/0/0/0
Kobayashi (2022) [31]	Japan	P	233	77	75	233/0	118/115	213/20	0/116/72/23/0
Koga (2022) [32]	Japan	R	153	77	75	30/123	95/58	137/16	0/153/0/0/0
Kunkel (2021) [33]	Germany	R	97	67	75	25/72	60/37	NR/NR	54/33/NR/NR/NR
Kuroda (2017) [34]	Japan	R	895	72	65	659/236	460/435	NR/NR	0/NR/426/91/NR
Li (2015) [35]	USA	R	237	NR	75	237/0	104/133	144/93	6/1/108/82/40
Li (2020) [36]	China	R	157	74	70	30/127	98/59	106/51	134/0/0/23/NR
Macchini (2021) [37]	Italy	R	105	NR	75	58/47	49/56	NR/NR	NR/34/NR/NR/0
Ohwada (2022) [38]	Japan	R	96	78	75	96/0	61/35	83/13	0/51/31/14/0
Patel (2021) [39]	USA	R	363	64	75	54/309	189/174	NR/NR	269/94/0/0/0
Petrillo (2019) [40]	Italy	R	64	69	65	32/32	23/41	55/9	0/45/0/65/0
Pignon (2021) [41]	France	R	127	72	75	42/85	67/60	98/29	0/127/0/0/0
Prager (2021) [42]	Austria	P	299	70	70	137/162	168/131	282/17	0/299/0/0/0
Prejac (2022) [43]	Croatia	R	139	67	65	83/56	76/63	139/0	0/64/75/0/0
Rehman (2020) [44]	USA	R	73	73	65	73/0	38/35	NR/NR	0/73/0/0/0
Riedl (2021) [45]	Austria	R	455	67	70	NR/NR	268/187	409/46	158/297/0/0/0
Tabernero (2015) [46]	Spain	P	861	NR	65	365/496	502/359	NR/NR	0/431/430/0/0
Takeda (2021) [47]	Japan	R	128	NR	70	NR/NR	52/76	128/0	33/95/0/0/0
Tezuka (2022) [48]	Japan	R	151	64	65	75/76	79/72	151/0	151/0/0/0/0
Ulusakarya (2019) [49]	France	R	37	64	65	NR/NR	22/15	37/0	37/0/0/0/0
Vivaldi (2020) [50]	Italy	R	156	71	70	91/65	84/72	135/21	0/156/0/0/0
Von Hoff (2013) [51]	USA	P	861	63	65	365/496	502/359	NR/NR	0/431/430/0/0
Xie (2020) [52]	USA	R	606	74	65	606/0	332/274	343/107	88/67/137/99/215
Zong (2020) [53]	China	R	110	62	65	20/90	64/46	NR/NR	0/110/0/0/0

Abbreviations: BSC, best supportive care; ECOG, Eastern Cooperative Oncology Group performance status; FLX, FOLFIRINOX; Gem, gemcitabine-based monotherapy; GNP, gemcitabine plus nab-paclitaxel; R, retrospective; P, prospective; NR, not reported.

**Table 2:** Summary of prespecified meta-analyses.

Outcome/Comparison	No. of Studies	Pooled Effect (HR, 95% CI)	*p* Value (Overall Effect)	*I* ^2^	Heterogeneity *p* Value
CTx vs. BSC: OS [17,19,20,21,22,28,29,34,35]	9	0.46 (0.39–0.54)	<0.001	18%	0.28
Age subgroup (older vs. younger): OS [14,15,16,17,18,21,23,24,25,26,27,30,32,33,34,35,36,37,38,39,40,41,42,43,44,45,46,47,48,49,50,51,52,53]	34	1.00 (0.99–1.02)	0.76	23%	0.12
Age subgroup (older vs. younger): PFS [14,26,30,32,36,41,42,43,48,50,51]	11	0.96 (0.86–1.07)	0.44	10%	0.35
Combination CTx vs. monotherapy: OS [14,16,17,19,25,28,31,35,38,43,46,51,52]	13	0.66 (0.54–0.80)	<0.001	86%	<0.001
Combination CTx vs. monotherapy: PFS [14,25,31,38,43,46,51]	7	0.63 (0.53–0.74)	<0.001	30%	0.20
FOLFIRINOX vs. gemcitabine/nab-paclitaxel: OS [17,22,24,25,30,39,45,47]	8	0.98 (0.90–1.05)	0.52	60%	0.01
FOLFIRINOX vs. gemcitabine/nab-paclitaxel: PFS [45,47]	2	0.97 (0.92–1.02)	0.24	0%	0.93

Abbreviations: HR, hazard ratio; CI, confidence interval; CTx, chemotherapy; BSC, best supportive care; OS, overall survival; PFS, progression-free survival.

## Data Availability

All data analyzed in this study are derived from published articles included in the systematic review and from information available in the tables and figures of this manuscript. Additional details are available from the corresponding author upon reasonable request.

## Supplementary Materials

The following supporting information can be downloaded at: https://www.mdpi.com/article/10.3390/jcm15062254/s1, Figure S1: Funnel plots for publication bias assessment across major comparisons in the meta-analysis; File S1: PRISMA Checklist.

## Abbreviations

The following abbreviations are used in this manuscript:

BSC	Best supportive care
CI	Confidence interval
FLX	FOLFIRINOX
GNP	Gemcitabine plus nab-paclitaxel
HR	Hazard ratio
I2	Inconsistency index (I-squared statistic)
MOOSE	Meta-analysis Of Observational Studies in Epidemiology
OS	Overall survival
PDAC	Pancreatic ductal adenocarcinoma
PFS	Progression-free survival
PICOS	Population, Intervention, Comparison, Outcomes, and Study design
PRISMA	Preferred Reporting Items for Systematic Reviews and Meta-Analyses
