# Supplementary figures and images for: Chemotherapy for Older Adults with Locally Advanced or Metastatic Pancreatic Ductal Adenocarcinoma: A Systematic Review and Meta-Analysis
Source: J Clin Med. 2026 Mar 16;15(6):2254. doi: 10.3390/jcm15062254 (PMC13026222; doi:10.3390/jcm15062254)

## Slide 1
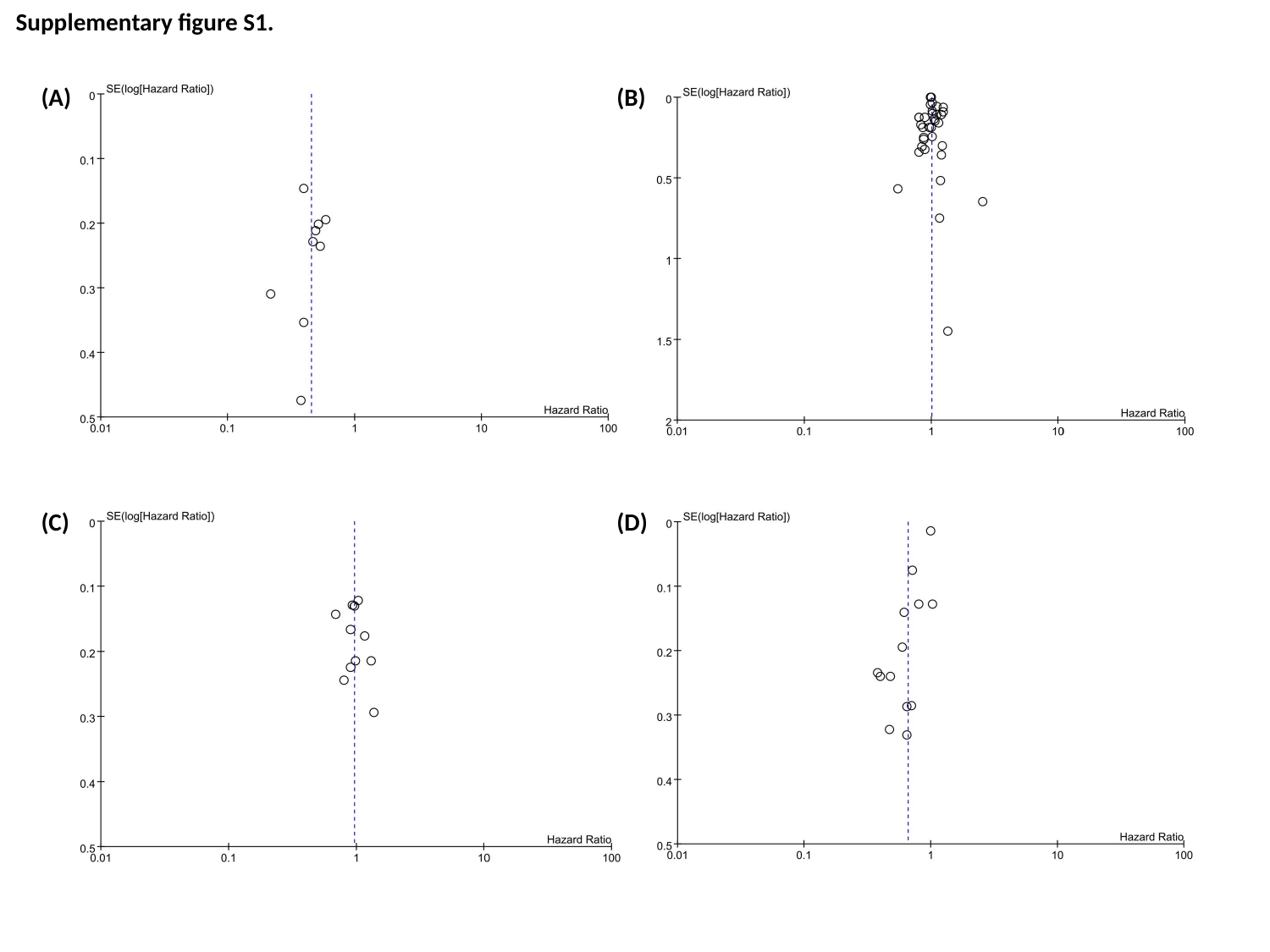

Supplementary figure S1.
(A)
(B)
### Chart
| Category |
|---|(C)
(D)

## Slide 2
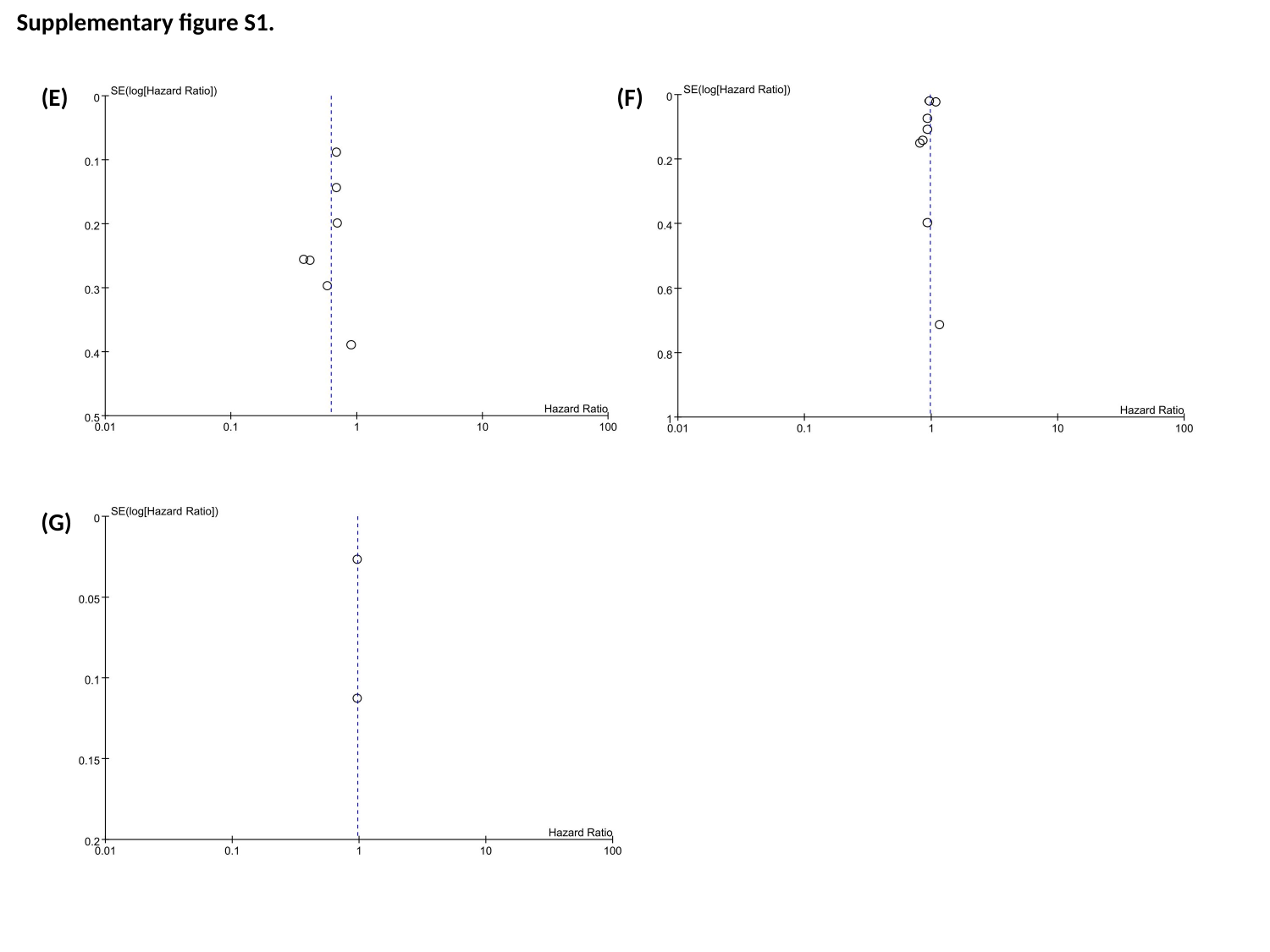

Supplementary figure S1.
(E)
(F)
(G)

Supplement: Supplementary file 1 [file jcm-15-02254-s001.zip › jcm-4163951-supplementary.pptx]
